# Clinical efficacy of local targeted chemotherapy for triple-negative breast cancer

**DOI:** 10.2478/v10019-011-0014-7

**Published:** 2011-04-23

**Authors:** Jinsong He, Xianming Wang, Hong Guan, Weicai Chen, Ming Wang, Huisheng Wu, Zun Wang, Ruming Zhou, Shuibo Qiu

**Affiliations:** The Center of Diagnosis and Treat of Breast Disease, The Second People’s Hospital of Shenzhen City, Shenzhen, P. R. China

**Keywords:** triple-negative breast cancer, targeted chemotherapy, prognosis

## Abstract

**Background:**

The aim of the study was to evaluate the clinical efficacy of superselective intra-arterial targeted neo-adjuvant chemotherapy in the treatment of estrogen receptor (ER)-negative, progesterone receptor (PR)-negative, and human epidermal growth factor receptor 2 (HER2)-negative (triple-negative) breast cancer.

**Patients and methods.:**

A total of 47 triple-negative breast cancer patients (29 at stage II, 13 at stage III and 5 at stage IV) were randomly assigned to two groups: targeted chemotherapy group (n=24) and control group (n=23). Patients in the targeted chemotherapy group received preoperative superselective intra-arterial chemotherapy with CEF regimen (C: cyclophosphamide [600 mg/m^2^]; E: epirubicin [90 mg/m^2^]; F: 5-fluorouracil [600 mg/m^2^]), and those in the control group received routine neoadjuvant chemotherapy with CEF. The duration of the treatment, changes in lesions and the prognosis were determined.

**Results:**

The average course of the treatment was 15 days in the targeted chemotherapy group which was significantly shorter than that in the control group (31 days) (*P*<0.01). The remission rate of lesions was 91.6% in the targeted chemotherapy group and 60.9% in the control group, respectively. Among these patients, 9 died within two years, including 2 (both at IV stage) in the targeted chemotherapy group and 7 (2 at stage II, 4 at stage III and 1 at stage IV) in the control group.

**Conclusions:**

As an neoadjuvant therapy, the superselective intra-arterial chemotherapy is effective for triple-negative breast cancer, with advantages of the short treatment course and favourable remission rates as well as prognoses.

## Introduction

Triple-negative breast cancer refers to breast cancers negative for estrogen receptor (ER), progesterone receptor (PR), and human epidermal growth factor receptor 2 (HER2), accounting for about 15% of breast cancers of all types.^1^–^4^ Triple-negative breast cancer progresses rapidly and is susceptible to distant metastasis due to the lack of the effective targeted endocrine therapy and anti-HER-2 therapy, resulting in a high mortality. Patients with triple-negative breast cancer have a high risk for death, and no effective treatment has been developed yet.^4^–^6^ The polymerase inhibition might be an effective treatment for triple-negative breast cancer, but it’s still under study.^7^ Since April 2006, superselective intra-arterial targeted neoadjuvant chemotherapy has been applied in our hospital for the short-course treatment of triple-negative breast cancer. The clinical efficacy of this method in the treatment of triple-negative breast cancer and its effect on the prognosis of this disease were analysed herein.

## Patients and methods

### General data

A total of 47 patients with triple-negative breast cancer were recruited from April 2006 to March 2010 from the Center of Breast Disease of our hospital. All patients underwent core needle biopsy, and the pathological examination was conducted to detect the ER, PR and HER-2, and the diagnosis of triple-negative breast cancer was confirmed. The cancers were at stage IIA–IV and primary invasive breast cancer. The median age was 41 years (range: 26–58 years).

These patients were randomly assigned into a targeted chemotherapy group and a control group. Before chemotherapy, all patients underwent ultrasonography of bilateral breasts, bilateral armpits and the liver, chest radiography and systemic bone scanning. For the enlarged lymph nodes, the fine needle aspiration biopsy was done to detect whether there was metastasis. There were 24 patients in the target chemotherapy group with a median age of 42 years (range: 28–58 years). Of them, 5 and 19 were negative and positive for regional lymph node metastasis, respectively. In respect of the TMN stage, there were 6, 8, 4, 2 and 4 cases at the stage of IIA, IIB, IIIA, IIIC and IV, respectively. In the control group (n=23), the median age of patients was 41 years (range: 26–55 years). Of them, 4 and 19 were negative and positive for regional lymph node metastasis, respectively. In respect of the TMN stage, there were 6, 9, 4, 3 and 1 cases at the stage of IIA, IIB, IIIA, IIIC and IV, respectively. There were no significant differences in the age or stage of cancers between both groups (P=0.643 and 0.514, respectively).

### Neoadjuvant chemotherapy

#### Targeted chemotherapy group

**FIGURE 1.**
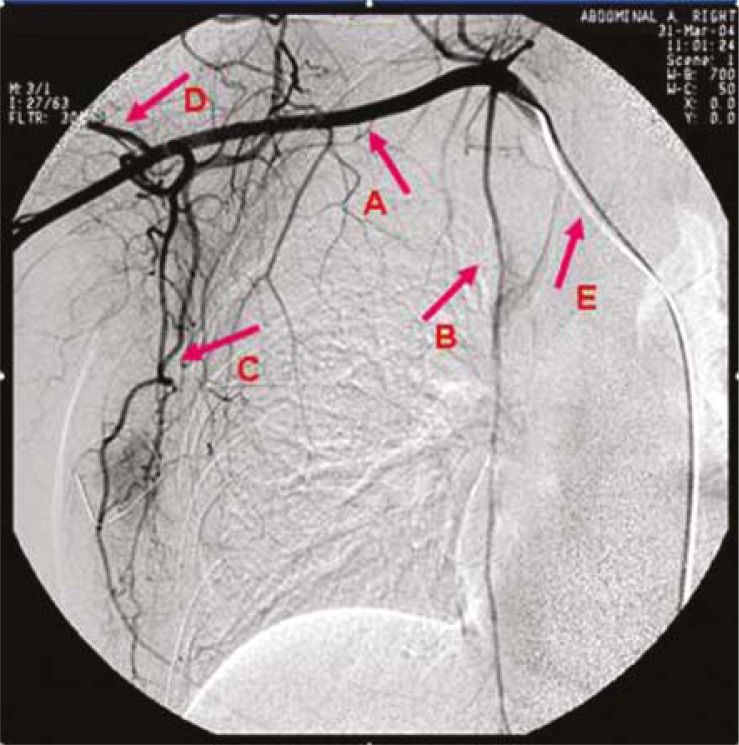
Blood vessel network under digital subtraction angiography. A = subclavian artery; B = internal mammary artery; C = lateral thoracic artery; D = circumflex scapular artery; E = Catheter

**FIGURE 2.**
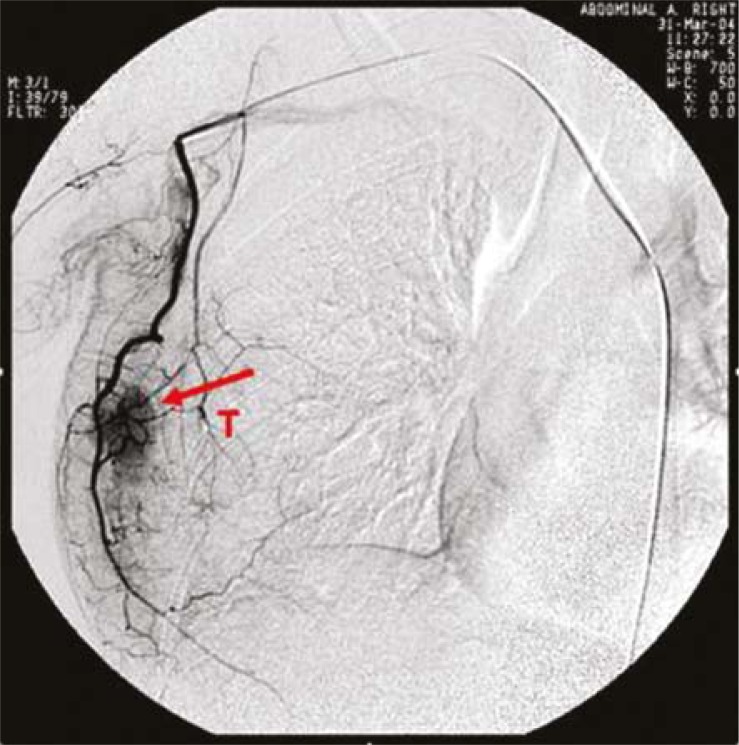
Breast cancer and its feeding arteries. T = tumour

Before surgery, superselective intra-arterial targeted neoadjuvant chemotherapy was performed.^8^,^9^ The femoral artery is punctured with a Seldinger needle and a 5-6F catheter was inserted into ipsilateral subclavian artery. The blood vessel network of breast, feeding arteries of the cancer and blood supply to the cancer were presented under angiography (Figures 1, 2). The superselective catheterization was performed into a main feeding artery of the cancer, followed by infusion of half amount of the drug, and then into the main feeding artery (lateral thoracic artery or internal mammary artery) of the breast followed by infusion of 25% of the drug, and finally into the distal end of the cross between subclavian artery and vertebral artery followed by the infusion of the remaining drug (involving the whole blood vessel network of the breast and blood vessels in the armpit). Then, the blood flow of brachial artery was blocked by pneumatic tourniquet avoiding the entry of drug to the brachial artery. The duration of the whole perfusion was 3–5 h, and all patients treated with CEF regimen (C: cyclophosphamide [600 mg/m^2^]; E: epirubicin [90 mg/m^2^]; F: 5-fluorouracil [600 mg/m^2^]). A cycle lasted 21 days.

#### Control group

Before surgery, routine intravenous chemotherapy was carried out. All patients received chemotherapy with CEF regimen (C: 600 mg/m^2^ at day 1; E: 90 mg/m^2^ at day 1; F: 600 mg/m^2^ at day 1). A cycle lasted 21 days.

#### Surgery and post-operative adjuvant therapies

After 1–2 courses of chemotherapy, all patients received surgery. In the targeted chemotherapy group, 6 patients received classical radical mastectomy (Halsted), 10 modified radical mastectomy, 4 breast-conserving surgery and 4 palliative resection of breast cancer. In the control group, 9 patients received classical radical mastectomy (Halsted), 7 modified radical mastectomy, 2 breast-conserving surgery and 5 palliative resection of breast cancer. After surgery, adjuvant chemotherapy with previous regimen was performed for a total of 6 courses. In addition, 20 patients experienced radiotherapy. Clinical manifestations were observed during the study including changes in lesions, days of the treatment, complications (adverse effects were diagnosed according to Criteria for Acute and Subacute Toxicity by WHO), and the post-operative follow up was also carried out.

#### Statistical analysis and ethical consideration

Comparisons of means between two groups were done with t test and one way analysis of variance was used to analyse the difference in the means between multiple groups. A pairwise comparison was done with q test. The statistical analysis was performed with SPSS 10.0 statistic software package. A value of P < 0.05 was considered statistically significant. The study was carried out according to the Helsinki Declaration.

## Results

### Short-term efficacy and changes in TNM stage and N stage after treatment

**FIGURE 3.**
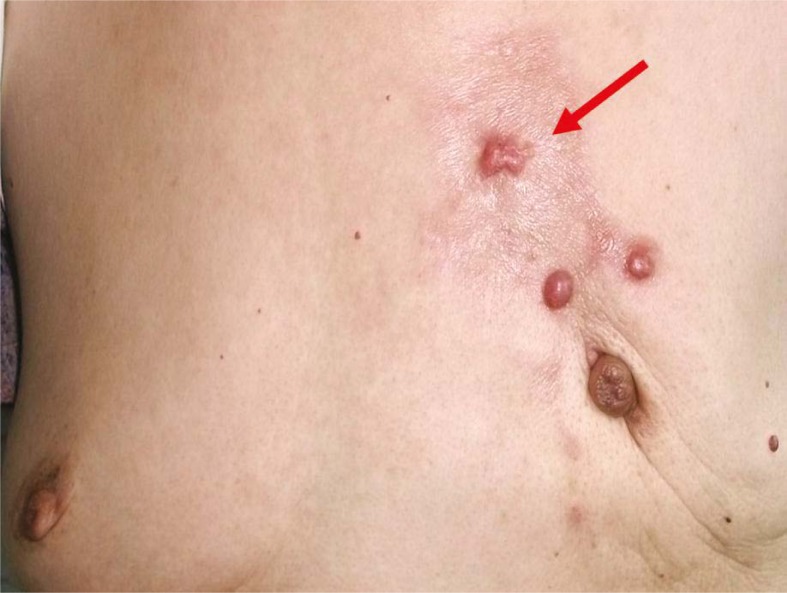
Lesions before targeted chemotherapy.

**FIGURE 4.**
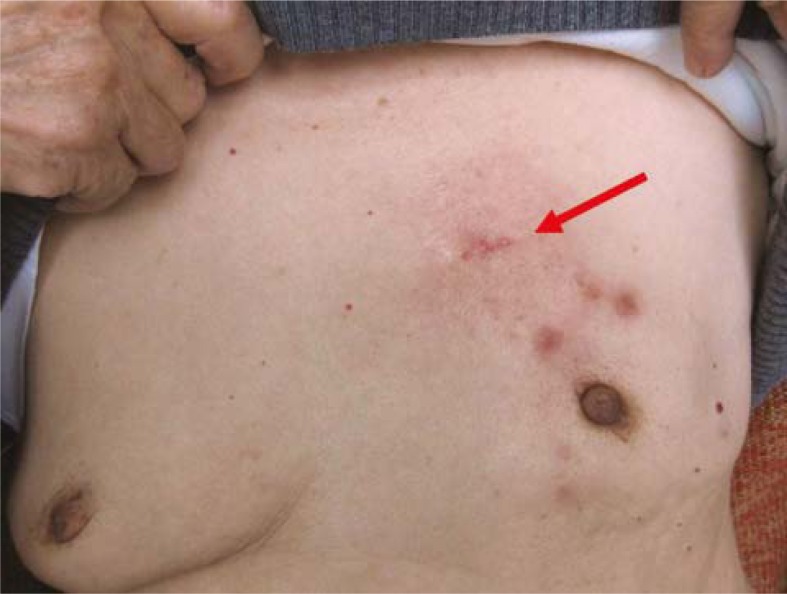
The lesions were markedly improved after targeted chemotherapy.

**FIGURE 5.**
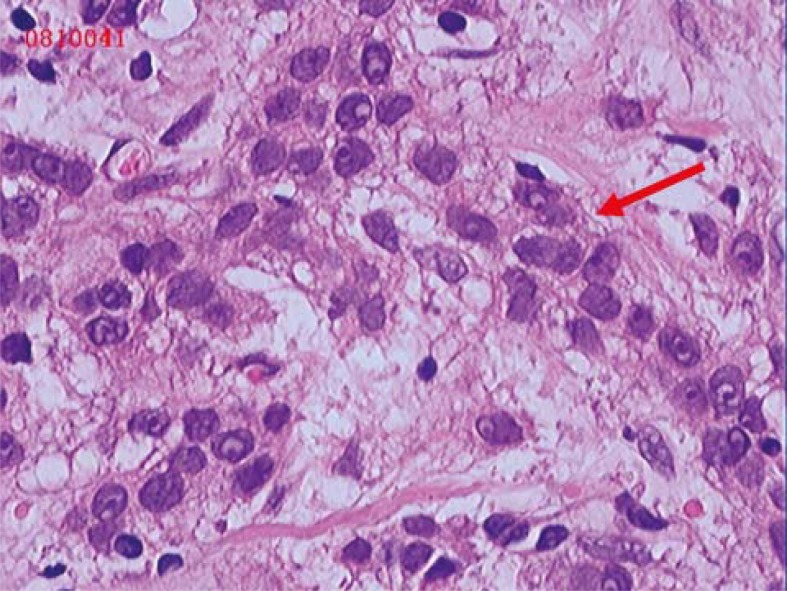
Pre-operative aspiration biopsy and pathological examination showed invasive ductal carcinoma (HE ×200).

In the targeted chemotherapy group, changes of local lesions were observed as early as 3 days after chemotherapy. Oedema at the local lesion was attenuated and accompanied by the occurrence of fold, the superficial varicosity was alleviated, and the adherence between cancers and chest wall was improved. The mass size was decreased, and the lesion was softened and could be moved. The exudate in the ulcerated site was reduced, and the skin colour was changed into brownness (Figures 3, 4). In the targeted chemotherapy group, nine patients achieved a clinical complete remission (cCR), thirteen patients achieved a partial remission (PR), one patient achieved state disease (SD), one patient achieved progress disease (PD); four patients achieved a pathological complete remission (pCR), and the remission rate (RR) was 91.67 % (22/24) (Figure 5∼6). Residual carcinoma *in situ* was noted in 1 patient. The down-staging rate was 62.50% (15/24). In the control group, the changes of local lesions were observed at 10 days after chemotherapy. The mass was softened and tumour size was reduced, which was more obvious 30 days after chemotherapy. In the control group, there were 3, 11, 7, 2, and 1 patient with cCR, PR, SD, PD and pCR, respectively, and the RR was 60.87% (14/23). The down-staging rate was 39.13% (9/24). In the targeted chemotherapy group, of the 19 patients with an axillary lymph node metastasis, no metastasis was found in regional lymph nodes in 7 patients after surgery. In the control group, of 19 patients with an axillary lymph node metastasis, no metastasis was found in regional lymph nodes in 7 patients after surgery. The negative change ratio in a lymph node metastasis was 47.37% (7/19) in the targeted chemotherapy group and 26.31% (5/19) in the control group. The statistical analysis showed there were significant differences in the down-staging rate and negative change ratio between both groups (P=0.023 and 0.041, respectively). The efficacy of targeted chemotherapy was superior to traditional chemotherapy (P=0.018). The targeted chemotherapy was also superior to traditional chemotherapy in terms of cCR and pCR (P=0.016 and 0.018).

#### Course of treatment

In the targeted chemotherapy group, 18 patients and 6 patients received surgery after 1 course and 2 courses of chemotherapy, respectively. The mean duration of the treatment was 15 days. In the control group, 8 patients and 15 patients received surgery after 1 course and 2 courses of chemotherapy, respectively. The mean course of the treatment was 31 days. A statistical analysis showed the mean course of the treatment in the targeted chemotherapy group was shorter than in the control group (*P*<0.01).

#### Toxicity of treatment

The grades of chemotherapy toxicity were 0 in 14 patients (58.3%), I in 7 patients (29.2%), II in 3 patients (12.5%) in the targeted chemotherapy group; 0 in 12 patients (52.2%), I in 7 patients (30.4%), II in 4 patients (17.4%) in the control group. There was no significant difference in the degree of drug toxicity between two groups (*P*>0.05). The toxicity of degree 0–1 can resolve spontaneously, and that of degree II can resolve within 1 week after the general treatment.

#### Follow up

The follow up period was 8–48 months after the surgery. A total of 41 patients (87.23%) completed the follow up and 6 were lost to the follow up. There were 22 patients (91.67%) in the targeted chemotherapy group and 19 patients (82.61%) in the control group completing a follow up. A total of 9 patients died within 2 years including 2 (at stage IV) in the targeted chemotherapy group and 7 in the control group (2 at stage II, 4 at stage III and 1 at stage IV).

## Discussion

With the understanding of the genomic profile of breast cancer, the molecular biological types of breast cancer can be determined. Breast cancer of different subtypes may have significantly distinct prognosis, different therapeutic strategies and marked difference in survival.^10^–^12^ Patients with triple-negative breast cancer cannot benefit from the targeted endocrine therapy and anti-HER-2 treatment. Therefore, for such patients, the only alternative strategy is chemotherapy as an adjuvant therapy of surgery and radiotherapy. Studies have demonstrated triple-negative breast cancer is more sensitive to chemotherapeutic drugs than breast cancer of other subtypes.^13^,^14^ However, the prognosis of triple-negative breast cancer is very poor and it is susceptible to recurrence, which may be contributed to the low pathological remission rate of triple-negative breast cancer. Therefore, in enhanced chemotherapy, the pathological remission rate is a critical factor in improving the prognosis of triple-negative breast cancer.

The sensitivity to chemotherapeutic drugs and local concentration are two crucial factors determining the fate of cancer cells (especially the tumour stem cells) and the subsequent pathological remission rate.^15^ Traditionally, the pre-operative chemotherapy is carried out through intravenous infusion. Under this condition, the effective concentration of chemotherapeutic drugs in local lesion is relatively low and, therefore, the effectiveness will be achieved after prolonged courses of the treatment. In addition, intravenous chemotherapy may result in drug resistance of cancer cells. Nevertheless, in the intra-arterial chemotherapy, the effective concentration of chemotherapeutic drugs in local lesion, surrounding tissues and lymph nodes is significantly elevated.^16^,^17^ Previously, the blood supply of breast cancer was considered to be mainly from internal mammary artery. Therefore, in superselective intra-arterial chemotherapy, the chemotherapeutic drugs were infused into internal mammary artery. However, in recent studies^18^–^20^, results revealed the lateral thoracic artery was the dominant feeding artery of breast cancer followed by thoracic artery and sub-scapular artery. At the same time, the metastasis of breast cancer is done through ipsilateral axillary lymph nodes which are supplied by thoracic artery and subscapular artery. Therefore, the optimal target in superselective intra-arterial chemotherapy should be the blood vessel network in the breast cancer and the axillary arteries^8^ and the area supplied by these blood vessels is also called target region. In the present study, superselective intra-arterial chemotherapy was performed and the effective concentration of chemotherapeutic drugs in the local target region was dramatically increased in unit time, resulting in increased time-density/intensity. Therefore, the primary lesion and surrounding potential lesions as well as lymph nodes were effectively chemically treated which further increased the pathological remission rate and reduced the local recurrence and the risk for distant metastasis.

The favourable sensitivity to chemotherapeutic drugs but poor prognosis of triple-negative breast cancer may be contributed to the difficult detection of local skin metastases, lymph node metastases or even distant micrometastases. In traditional chemotherapy, the effective concentration in local lesion is relatively low in unit time and subsequently the cancer cells can not be completely killed, which, however, may lead to drug resistance of cancer cells affecting the efficacy of chemotherapy. Our results showed the advantages of targeted chemotherapy in the increased efficacy, down-staging, improved lymph node metastasis, cCR, and pCR over traditional chemotherapy (P<0.05). Furthermore, the mean course of targeted chemotherapy was only 15 days which was also shorter than that in the control group (31 days). These results revealed the superselective intra-arterial chemotherapy could increase the local effective concentration of chemotherapeutic drugs, possess favourable efficacy demonstrated by the decrease in tumour size and rapidly control the disease progression, which are critical for the killing of cancer cells as soon as possible.

Pre-operative superselective intra-arterial chemotherapy, on one hand, can increase the local effective concentration after the regional infusion, and more cancer cells can be killed when completely contacting with drugs. As a result, the tumour could be shrunk in a large scale. Therefore, the man-made spread of cancer cells during operations and post-operative recurrence as well as metastasis are prevented. On the other hand, the infused drugs are transferred into the systemic circulation. Therefore, not only local chemotherapy but systemic chemotherapy is carried out. In the present study, there was no significant difference in the side effects of chemotherapy between both groups, which suggested the concentration of drugs in the systemic circulation was also comparative with that in traditional chemotherapy. This effect is critical for the control of subclinical lesion and benefits for the complete remission in pathology. Moreover, the mortality in the targeted chemotherapy was lower than that in the control group, which also demonstrated the advantage of targeted chemotherapy in improving the prognosis of triple-negative breast cancer.

## Figures and Tables

**FIGURE 6.**
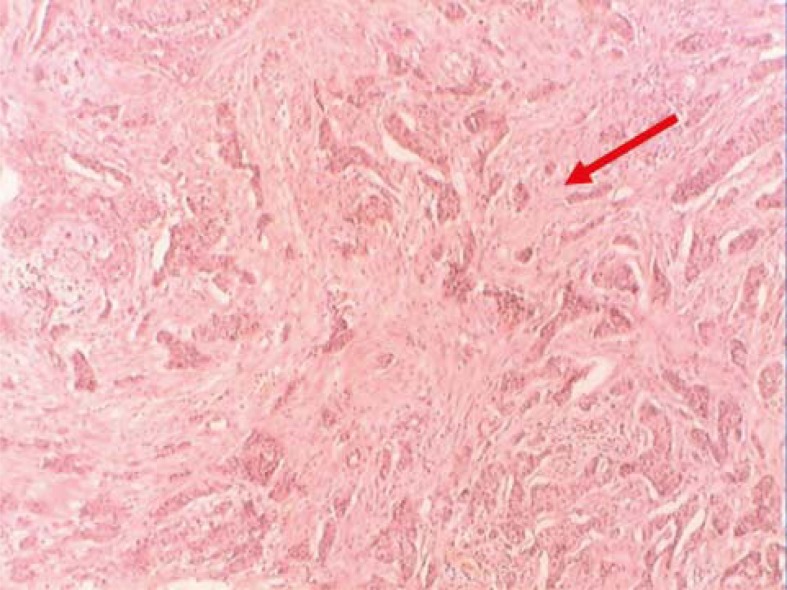
The residual cancer nests were not found after targeted chemotherapy (H&E×200).
